# Gonococcal polyarthritis with sternoclavicular joint involvement in pregnant woman: a case report

**DOI:** 10.11604/pamj.2014.17.242.4181

**Published:** 2014-03-31

**Authors:** Imane El Mezouar, Latifa Tahiri, Faiza Lazrak, Khadija Berrada, Taoufik Harzy

**Affiliations:** 1 Rheumatology Department, Medical School, Sidi Mohammed Ibn Abdellah University, Hassan II University Hospital, Fez, Morocco

**Keywords:** Gonococcal polyarthritis, sternoclavicular joint, pregnancy

## Abstract

Pregnancy is one of conditions that increase the risk of gonococcal arthritis which result from blood dissemination of neisseria gonorrhoeae. A 20-year-old africain female patient (in the second trimester of pregnancy), was admitted to hospital because of fever, asymmetric joint swollen affecting the hands, wrists, left ankle, and right sternoclavicular joint. Laboratory findings (erythrocyte sedimentation rate was 117 mm in first hour, The serum C-reactive protein level was 152 mg/L) the gram stain of genital sample was positif of neisseria gonorrhoeae and trichomonas vaginalis. With antibiotics, outcome of pregnancy was timely and uneventful. Patients should be educated about the mode of transmission of gonorrhea. Sexual partners should also be treated to prevent dissemination and gonococcal re-infection.

## Introduction

Gonococcal arthritis is a common, well described entity; it tends to affect the knees, wrists, ankles and finger joints. Sternoclavicular joint involvment is rare. We present an unusual case of gonococcal arthritis identified in pregnant woman affecting sternoclavicular joint.

## Patient and observation

**Consent statement**: Written informed consent was obtained from the patient's legal guardian(s) for publication of this case report and any accompanying images. A copy of the written consent is available for review by the Editor-in-Chief of this journal.

A 20-year-old, divorced africain female patient, with multiple sexual partners, low socio-economic status, G1P0, 28 weeks pregnant woman. Her past history was marked by offensive yellow vaginal discharge one month before she was admitted to hospital with a 2 day history of fever and asymmetric joint swollen affecting the hands, wrists, left ankle, and right sternoclavicular joint. The physical examination showed a temperature of 40°, painful swelling of the right sternoclavicular joint, the wrists, right knee, tenosynovitis of Extensor areas of the hand, and hemorrhagic pustules over the dorsum of the feet. Complete blood count showed leukocytosis (white blood cell count of 10360 cells/mm^3^ with 80% neutrophils). Erythrocyte sedimentation rate was 117 mm 1hour, The serum C-reactive protein level was 152 mg/L, A urine sample contained 1 930 000 leukocytes/mm^3^ with trichomonas vaginalis in culture.

**Figure 1:**
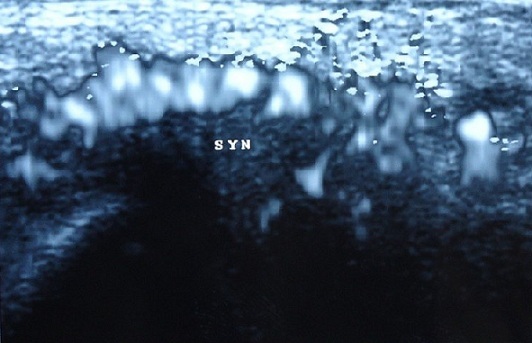
Active synovitis in Doppler with erosion of right sternoclavicular joint

Vaginal swab was positive for Neisseria gonorrhoea. Complement concentrations were normal, TPHA-VDRL was non-reactive, B Hepatitis Antigen. Human immunodeficiency virus (HIV) antibody was negatif. Ultrasound of swelling joint showed: Power Doppler enhanced tenosynovitis of extensor pollicis brevis and abductor pollicis longus, active synovitis of radio carpal joint and metacarpophalangeal joint. Active synovitis in Doppler with erosion of right sternoclavicular joint (Figure 1). Antibiotic treatment was started with intravenous cephalosporin (ceftriaxone: 1g/day) during two days. Oral therapy by cefixime 400 mg twice a day over 7 days, Ténonitrozole 250 twice a day to treat trichomonas vaginalis and azithromycin (a single 1 g oral dose) for chlamydia. Improvement in fever and joint involvement obtained within a few days (CRP 6). The outcome of pregnancy was timely and uneventful.

## Discussion

Gonococcal infection is still prevalent in developing countries and mainly observed in sexually active young adults. If gonorrhea is contracted during pregnancy, there is an increased risk of dissemination (DGI) [1]. Gonococcal arthritis develops in approximately 42-85% of patients with DGI [2]. It may be classified into « bacteraemic form » and « suppurative form », but many patients have features of both [3]. In bacteraemic form, clinical features include migratory polyarthralgia (knees, elbows and ankles), moderate fever, chills, vesicular or pustular lesions, tenosynovitis (50 to 60% of cases of gonococcal arthritis [4]) particularly extensor tendon of the hands, wrists, fingers, toes and ankles. Tenosynovitis and polyarthralgia may be related to autoimmune abnormalities induced by N.gonorrhoeae. In « suppurative form » : septic arthritis (joint swelling and effusion) is frequent: it occurs in 50% of DGI patients. Arthritis is frequently monoarticular. The most commonly involved joint are the knees, wrists, ankles and fingers. Hip involvement is rare [5]. Septic discitis [6] and sternoclavicular arthritis are even rare [7, 8]. A destructive arthritis of sterno clavicular joint was described [4].

Risk factors of gonococcal arthritis are : Female sex, Pregnancy, Menstruations, Multiple sexual partners, Low socio-economic status, Intravenous drug use, Complement deficiency, HIV infection, Systemic lupus erythematosus, Gonococcus strain characteristics (Protein 1A serotype, Lack of protein II) [3]. Hospitalization of gonococcal arthritis patients is recommended to confirm diagnosis, search systemic complications including endocarditis, myocarditis, hepatitis (Fitz -Hugh -Curtis syndrome), meningitis and to start antibiotic treatment [3]. N. gonorrhoeae is isolated from blood and synovial cultures in 50 % of gonococcal arthritis patients. When a synovial effusion is present, it should be aspirated. The leukocyte count in synovial fluid is inflammatory, usually in the range of 10 000 to 100 000 cells/mm^3^ [3]. In patients with purulent joint effusions, synovial fluid culture is often positive with negative blood cultures [9]. Gonococcal arthritis responds well to antibiotics and prognosis is good when appropriate therapy is quickly initiated. Destructive arthritis may be observed in HIV patients or in chronic infections due to inappropriate treatment. Third-generation cephalosporins are the first choice treatment [10], such as ceftriaxone, (1 g IM/IV), ceftizoxime (1g IM/IV every 8h) and cefotaxime (1g IV every 8h). If the woman is allergic to β lactam drugs, spectinomycin, 2 g IM every 12 h may be used. Spectinomycin and ceftriaxone can be used in pregnant women. Parenteral antibiotics should be continued until symptoms have improved for 24-48 h. Oral therapy may then be prescribed to complete 7 days of antibiotic. Cefixime 400 mg twice a day, ciprofloxacin 500 mg twice daily, can be given per os, Ciprofloxacin is contraindicated during pregnancy.

Repeat cultures of all positive sites at least 5 days after the last antibiotic dose are recommended. Infected joints should be aspirated to monitor the decrease in leukocyte count of synovial fluid. Saline lavage can also be used. Surgical treatment is exceptionally indicated [3]. If chlamydial infection is identified, tetracycline or doxycycline for 7 days (not allowed in pregnant women) or azithromycin (a single 1 g oral dose) should be started. Patients should be educated about the mode of transmission of gonorrhea, tested for HIV and syphilis initially and after 4-6 weeks. Sexual partners should also be treated to prevent dissemination and gonococcal re- infection [3].

## Conclusion

Education about the sexual mode of transmission of the disease and the means of preventing sexually transmitted diseases is an integral part of the treatment.
